# Supplementary material for: A machine learning approach to explore individual risk factors for tuberculosis treatment non-adherence in Mukono district
Source: PLOS Glob Public Health. 2023 Jul 3;3(7):e0001466. doi: 10.1371/journal.pgph.0001466 (PMC10317231; doi:10.1371/journal.pgph.0001466)
Supplement: Attachment — Submitted filename: Response to Reviewers.docx [file pgph.0001466.s001.docx]

Dear Dr. Collins (PhD),

Thank you for giving us the opportunity to submit a revised draft of our manuscript titled
*A machine learning approach to explore individual risk factors for tuberculosis treatment non-adherence in Mukono district* to PlosOne Journal of Global Health. We appreciate the time and effort that you and the reviewers have dedicated to providing your valuable feedback on our manuscript. We are grateful to the reviewers for their insightful comments on our paper. I have been able to incorporate changes to reflect most of the suggestions provided by the reviewers. I have highlighted the changes within the manuscript.

Here is a point-by-point response to the reviewers’ comments and concerns.

Comments from Reviewer 1

• Comment 1: *We have noticed that you have cited Table 5 in the manuscript file but there are no corresponding tables in the manuscript. Please amend your manuscript to include this table, noting that tables should not be uploaded as individual files*.

Response: Thank you for pointing this out. We agree with this comment. Therefore, we have added two tables in the results section (table 1, line number 369 and table 2, line number 373). Both tables are highlighted in yellow.

• Comment 2: *I have a concern as to your choice of the SVM model on one hand and Accuracy on the other hand. 5 models were used to train your AI algorithm and 5 performance matrixes were assessed. The following conclusion seems to misrepresent the outcomes unless I am missing something: "Of the five developed and evaluated models, SVM performed the best with an accuracy of 91.28% compared to RF (89.97%), LR (88.30%), ANN (88.30%), and AdaBoost (91.05%) respectively."*

“*I would say of the five models evaluated, SVM had the highest accuracy (AdaBoost performed better than SVM when AUC is considered as evaluation parameter). Looking at all 5 evaluation parameters globally, AdaBoost is quite on par with SVM. The different evaluation parameters tell different stories. It may be good to explain clearly if one parameter is classed above the others (in this case, your abstract singles out Accuracy to mention superiority of the SVM model) and highlight the performance of other models where they may add value to the overall assessment”.*

Response: Thank you for pointing this out. We have, accordingly, revised this statement to incorporate the proposed changes in the results [375-378] section. Further, We have explained the briefly discussed the different machine learning performance metrics and their use case and the choice of accuracy as our metric of choice [line 289 - 305].

**Comments from Reviewer 2**

• **Comment 1:** *Introduction: I find this too long – provide rationale for predicting patients who are likely to be non-adherent. The sentence starting on line 58, "Treatment non-adherence...." breaks the flow with the next paragraph. Please revise to improve on the flow.*

**Response:** We agree with this and have incorporated your suggestion. We have revised the introduction section, improving the flow, making it shorter [lines 44 - 84] and providing the rationale for predicting patients who are likely to be non-adherent [lines 50 - 54].

• **Comment 2:** *Attributes included in development of model should reflect intended public health benefits of model. By the 6th month of treatment, there are a number of missed opportunities to address adherence issues, therefore including sputum collection at 6 months as a predictor of non-adherence is difficult to justify.*

**Response:** Our machine learning algorithm training was done using retrospective data to generate a model that quantifies the factors based on the primary data capture tool [health facility registers], predictive of treatment non-adherence. Certainly, these quantified factors could potentially identify a specific individual at risk of treatment non-adherence. Although the sputum test is not done during the treatment initiation, the results from our model could potentially serve as an early warning to the health worker that, if the other non-adherence factors identified by the model are present, special guidance should be taken to also inform the patient on the importance of completing all the sputum tests and most especially during months 5 and 6. This is explained in the discussion section [line numbers 459 - 467].

*How did you measure the primary exposure non-adherence? TB patients on treatment do not typically have facility files where self-adherence to drugs is documented. The assumption therefore is that you measured this from records in the Unit TB register. How did you approach this?*

**Response:** This was a surrogate measure as follows “any patient who did not visit the health facility for a treatment drug refill in 28 days as was instructed by the health worker”. In the registers, the clinicians/dispenser normally notes down the previous date for drug refill and when the patient comes for a drug refill, this is update. It is worth noting that the patient is verbally informed of the expected return date for drug refill. Also, that date is written on the patient paper-based card for reference. Thus, we simply abstracted this as recorded in the health facility registers. This is highlighted in manuscript lines 142 – 145.

• **Comment 3:** *In 1 - 2 sentences, describe Mukono district - location, catchment area to provide context to the challenges faced by TB patients in seeking care.*

**Response:** Thank you for this guidance. This has been updated in the study design and setting section [lines 146 - 149]

• **Comment 4:** *Study period overlaps with the COVID19 pandemic lockdown period. Could this have affected your model?*

Response:  Partly yes, However, based on our abstracted records (1st January 2019 to 31st December 2021), most of the patients were already initiated on and already completed treatment (usually 6-9 months). Furthermore, the few who were initiated late were partially affected by the lockdown (imposed on 25 March 2020). Fortunately, the government of Uganda put in place some mechanisms to ensure continuity of treatment for all patients with chronic illnesses including tuberculosis. These mechanisms included issuing movement permits to the patients, multi-month drug dispensing, and delivering the drugs to the patients' homes by the village health teams. Interestingly, this could inform our subsequent research by stratifying these modalities and studying whether they may have contributed to tuberculosis non-adherence.

• **Comment 5:** *Lines 479 – 481: Include important factors associated with non-adherence.*

**Response:** Thank you for this guidance. This has been revised [lines 401 - 405]

• **Comment 6:** *Reference for line 483 – 4, 487 – 492, 517 – 8, 519 - 21
Was there an attempt to stratify patients by HIV status? CPT status is only applicable to HIV positive individuals – therefore including in model building that involves HIV negative individuals is not proper.*

**Response:** Agreed, it would be better to stratify the patients by the HIV status. However, the advantage of the output model is that it caters for both the HIV negative and positive tuberculosis patients. Depending on the individual patient’s characteristics, a health worker will be guided on the next course of action.

• **Comment 7:** *Line 537, the sentence starting, “From our findings, ….,” does not build on the previous one that starts, “Comorbidity of tuberculosis and HIV/AIDS………….”. Please revise.*

**Response:** Thank you for this guidance. This has been revised [lines 489 - 495]

• **Comment 8:** *Conclusion: Line 549 – The conclusion that drug non-adherence is prevalent among TB patients is not supported by study findings. Is the second paragraph necessary?*

**Response:** Thank you for this guidance. The conclusion has been revised and the referred second paragraph removed. [lines 488 - 494]

• **Comment 9:** *Study limitations should be included in the discussion section, before the conclusion.*

**Response:** Thank you for this guidance. This has been revised. [Lines 502 - 521]

In addition to the above comments, all spelling and grammatical errors pointed out by the reviewers have been corrected. We look forward to hearing from you in due time regarding our submission and to respond to any further questions and comments you may have.

Sincerely,

Haron Gichuhi
