# Supplementary material for: A machine learning approach to explore individual risk factors for tuberculosis treatment non-adherence in Mukono district
Source: PLOS Glob Public Health. 2023 Jul 3;3(7):e0001466. doi: 10.1371/journal.pgph.0001466 (PMC10317231; doi:10.1371/journal.pgph.0001466)
Supplement: Attachment — Submitted filename: Response to Reviewers.docx [file pgph.0001466.s002.docx]

Dear Dr. Collins (PhD),

Thank you for giving us the opportunity to submit a revised draft of our manuscript titled
*A machine learning approach to explore individual risk factors for tuberculosis treatment non-adherence in Mukono district* to PlosOne Journal of Global Health. We appreciate the time and effort that you and the reviewers have dedicated to providing your valuable feedback on our manuscript. We are grateful to the reviewers for their insightful comments on our paper. We have been able to incorporate changes to reflect most of the suggestions provided by the reviewers. We have highlighted the changes within the manuscript.

Here is a point-by-point response to the reviewers’ comments and concerns.

Comments from Reviewer 2

• Comment 1: *I note that narratives that best fit in the discussion section still appear in your introduction. Please rectify. For example, lines 123-127 compare your study findings to previous those of studies - this should be presented in the discussion and not the introduction*.

Response: Thank you for pointing this out. We agree with this comment. Therefore, we have moved this paragraph to the discussion section (line 422-426) highlighted in yellow.

• Comment 2: *The sentence starting at line 50, "Furthermore...." is out of sync with the previous sentence that leads a presentation of factors associated with non-adherence. This paragraph could be better placed before the one presenting these factors, using an appropriate connector (such as moreover or yet at the beginning), with a linking sentence that initially points out the importance of studying factors associated with non-adherence and then leading into a sentence presenting some of the commonly studied factors*.

Response: Thank you for pointing this out. We have, accordingly, revised this statement to incorporate the proposed changes in the introduction [48-52] section.

• Comment 3: *Line 130, the sentence starting, "Unfortunately, based on our research..." should be re-written to, "Unfortunately, based on our literature search...". This is for the purpose of clarifying that the ideas being presented in this sentence are derived from a review of literature and not from the present study findings.*

Response: Thank you for pointing this out. We have, accordingly, revised this statement to incorporate the proposed changes [line 125]

• Comment 4: *Lines 86 - 136 are based summarised and used to enrich the discussion section. They do not fit well in the introduction.*

Response: Thank you for pointing this out. However, this section serves two purposes and has been commonly employed in previous articles utilizing a machine learning approach. First, it gives the reader detailed background on previous studies that have been conducted in tuberculosis using a machine learning approach. Secondly, it gives a justification of exploring a machine learning approach to solve the problem at hand.

• Comment 5: *Line 133: clarify which study is being referred to, in the sentence beginning, "This study...".*

Response: Thank you for pointing this out. We have, accordingly, clarified this by using the word “our” instead of “this study”.

• Comment 6: *Lines 99 - 105: which investigators are being referred to?*

Response: Thank you for pointing this out. We have, accordingly, corrected the sentence and pointed out that this were the “Iranian investigators” building on the paragraph 94 - 98.

• Comment 7:  *Lines 168 - 175 in data description are explanatory. Do we need them here?*

Response: Thank you for pointing this out. We have, accordingly, revised this statement to incorporate the proposed changes in the results.

• Comment 8:  *Revise and summarise paragraph starting at line 172*

Response: Thank you for pointing this out. We have, accordingly, revised this statement to incorporate the proposed changes in the results.

• Comment 9:  *What was the reason for excluding 162 records? Were they duplicates? A clear study flow diagram can help clarify.*

Response: Thank you for pointing this out. We have clarified this in the results sections [Line 343 -344]

• Comment 10: *Lines 184 -188: How did the additional column in the register facilitate elimination of duplicates? This may be unnecessary detail.*

Response: Thank you for pointing this out. We have, accordingly, deleted these details.

• Comment 11:  *Line 266: note and revise hanging sentence.*

Response: Thank you for pointing this out. We have, accordingly, revised this statement to incorporate the proposed changes [line 267 - 268].

• Comment 12:  *Lines 411 - 414 are misplaced. The first paragraph of the Discussion section should present a summary of the study results, yet these lines are explaining choice of metrics used (this should be moved lower in the discussion section). Lines 429-430 need to be moved to the 1st paragraph of the Discussion. You could refer to this resource for further insights:* [*https://plos.org/resource/how-to-write-conclusions/*](https://plos.org/resource/how-to-write-conclusions/)

Response: Thank you for pointing this out and the link for further guidance. We have, accordingly, revised this statement to incorporate the proposed changes in the results

• Comment 13:  *Thank you for including the study limitations. You could re-write these so that they don't undermine the credibility of your work.*

Response: Thank you for pointing this out. We have, accordingly, revised the entire limitation sections to incorporate the proposed changes [line 502 - 510].

In addition to the above comments, all spelling and grammatical errors have been corrected. We look forward to hearing from you in due time regarding our submission and to respond to any further questions and comments you may have.

Sincerely,

Haron Gichuhi
